# Deep learning-based framework for the distinction of membranous nephropathy: a new approach through hyperspectral imagery

**DOI:** 10.1186/s12882-021-02421-y

**Published:** 2021-06-19

**Authors:** Tianqi Tu, Xueling Wei, Yue Yang, Nianrong Zhang, Wei Li, Xiaowen Tu, Wenge Li

**Affiliations:** 1 Peking University China-Japan Friendship School of Clinical Medicine, Beijing, China; 2 Department of Nephrology, China-Japan Friendship Hospital, Beijing, China; 3 Department of Biomedical Engineering, Tsinghua University, Beijing, China; 4 School of Information and Electronics, Beijing Institute of Technology, Beijing, China; 5 Department of Nephrology, PLA Rocket Force Characteristic Medical Center, Beijing, China

**Keywords:** Membranous nephropathy, Idiopathic membranous nephropathy, Hepatitis B virus, Hyperspectral imagery, Deep learning

## Abstract

**Background:**

Common subtypes seen in Chinese patients with membranous nephropathy (MN) include idiopathic membranous nephropathy (IMN) and hepatitis B virus-related membranous nephropathy (HBV-MN). However, the morphologic differences are not visible under the light microscope in certain renal biopsy tissues.

**Methods:**

We propose here a deep learning-based framework for processing hyperspectral images of renal biopsy tissue to define the difference between IMN and HBV-MN based on the component of their immune complex deposition.

**Results:**

The proposed framework can achieve an overall accuracy of 95.04% in classification, which also leads to better performance than support vector machine (SVM)-based algorithms.

**Conclusion:**

IMN and HBV-MN can be correctly separated via the deep learning framework using hyperspectral imagery. Our results suggest the potential of the deep learning algorithm as a new method to aid in the diagnosis of MN.

## Background

Membranous nephropathy (MN) is one of the most common causes of nephrotic syndrome in adult patients across all ethnicities. It often leads to end-stage renal disease [1–3]. Current data confirmed that MN makes up approximately 20–40% of all patients with nephrotic syndrome worldwide [4, 5]. Most cases of MN are idiopathic membranous nephropathy (IMN), and the rest are secondary membranous nephropathy (SMN) attributed to various causes including systemic lupus erythematosus (SLE), malignancy, and hepatitis B virus infection [6]. More than 257 million individuals worldwide are estimated to suffer from chronic HBV infection, leading to nearly 1 million deaths annually [7]. HBV infection is common in China; approximately 20% of patients with HBV infection develop extrahepatic manifestations [8]. Among the extrahepatic manifestations related to HBV infection, HBV-related membranous nephropathy is a common outcome of HBV infection. The most crucial part of MN diagnosis is to distinguish between idiopathic or secondary disease based on the patients’ laboratory examination and renal biopsy outcome. These values determine the specific treatment decisions of the two and guide the long-term prognosis.

Conventional approaches to detecting glomerular hepatitis antigen/antibody include positive immunofluorescence outcomes and specific pathological features found in renal biopsy tissue using light and electron microscopy. In theory, IMN patients were associated with only immune complex deposition under the epithelial tissue and the thickening glomerular basement membrane (GBM). HBV-MN patients were characterized by immune complex deposited in multiple locations and podocyte proliferation besides the GBM lesions [1]. However, in our previous laboratory practice, we noticed that methods based on immunochemistry have a high number of false positives. Pathological features between IMN and HBV-MN on conventional bidimensional images obtained via light microscope are highly identical.

The morphological distinction between IMN and HBV-MN is subtle, and an alternative method is needed. Here, we propose a computer-based automatic identification method using a hyperspectral microscopy system. This is a sufficient supplementary approach to separate IMN and HBV-MN.

**Fig. 1:**
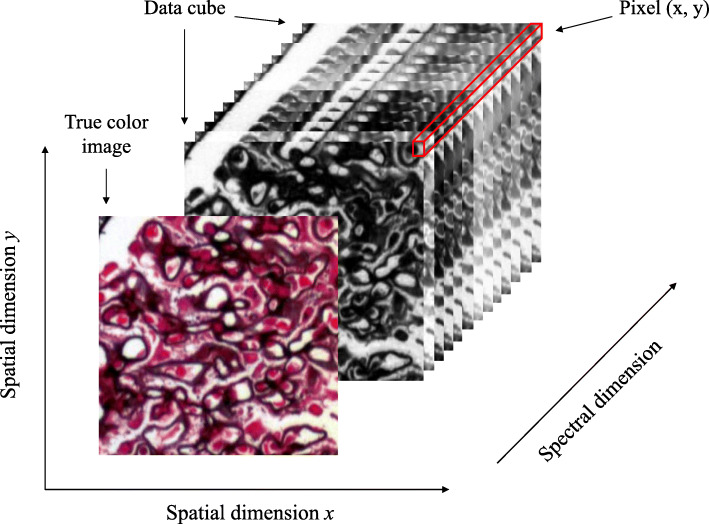
Example of a hyperspectral data cube from glomeruli. Each data cube contains two spatial dimensions and one spectral dimension

Hyperspectral imagery (HSI) is an advanced imaging technique capable of obtaining both spatial and spectral information of the target material; it is better than classic imaging techniques that only provide spatial information such as shape, size, and texture [9, 10]. Figure 1 shows the concept of hyperspectral data captured by HS imager. Each hyperspectral image can be visualized as a three-dimensional (3D) data cube consists of several two-dimensional (2D) grayscale images stacked together because of its intrinsic features. Every pixel of a hyperspectral image carries a specific value called a spectral signature that is determined by the material being observed. These features can then be extracted by deep learning algorithms for analysis. HS images contain more spatial information than traditional spectrometry method; it has incredible potential in pathology, cytogenetics, oncology, and clinical diagnosis [11–13].

According to the electromagnetic theory, different biochemical constituents usually have different spectral signatures [14]. IMN is a single-organ autoimmune disease, and the component of its immune complex (IC) deposition is connected to IgG4 initiation in pathogenesis [15], where the components of HBV-MN deposition are intimately related to the hepatitis B virion. The different pathological changes of IMN and HBV-MN lead to different spectral signatures, allows us to identify their distinction using HSI. Since its first introduction into biomedical use in the 1990s, HSI has shown excellent potential for noninvasive disease diagnosis and surgical guidance [9].

This is the first study using hyperspectral characteristics to discriminate between different IMN from HBV-MN in adult patients with membranous nephropathy. The hyperspectral image makes patient distinction straight forward.

## Methods

### Patient recruitment

We retrieved 20 patients diagnosed with membranous nephropathy by clinical data and renal biopsy in the China-Japan Friendship Hospital from July 2019 and September 2019, including 10 IMN patients and 10 HBV-MN patients. The inclusion criteria of the IMN group were MN patients with unclear etiology and glomerular lesions limited to the immune complex deposited under the epithelial and thickening glomerular basement membrane. The HBV-MN group had the following criteria: (1) serum HBV markers positive; (2) excluded other causes attributed to secondary renal disease (SLE, drugs, toxins, other infections, or malignancy); and (3) presence of detectable HBV-related antigen or antibody in renal biopsy tissue. This last criterion is the most fundamental and indispensable rule of all of those listed above. In all of the cases, MN was accompanied by other pathological patterns, and diabetic nephropathy and IgA nephropathy were ruled out. Demographic and clinical parameters of the MN patients are shown in Table 1.

**Table 1:** Demographic and clinical parameters of the 20 patients

Characteristic	IMN (***n*** = 10)	HBV-MN (***n*** = 10)
Age (years)	47.6 ± 14.6	50.3 ± 11.0
Men, *n* (%)	6 (60%)	7 (70%)
Proteinuria (g/24 h)	4.88(2.00, 6.95)	3.74(3.03, 5.36)
Albumin (g/L)	30(25, 39)	30(25, 31)
Serum creatine (μmol/l)	66.5(52.2, 83.6)	75.5(55.2, 98.7)
BUN (mmol/l)	4.67(3.34, 5.66)	4.85(3.73, 6.63)
Cholesterol (mg/dl)	6.34(5.13, 8.50)	7.46(6.23, 8.94)
PLA2R-ab (Ru/ml)	29.9(18.0, 56.8)	7.6(4.8, 15.9)

*Abbreviations*: *IMN* idiopathic membranous nephropathy, *HBV-MN* hepatitis B virus-related membranous nephropathy, *BUN* blood urea nitrogen, *PLA2R* M-type phospholipase A2 receptor

### Sample description

We collected glomerular characteristics for hyperspectral analysis with routinely processed renal biopsy tissue. Each sample was stained with hematoxylin-eosin (HE), periodic acid-Schiff (PAS), Masson’s trichrome, and Jones’s silver. All renal tissue samples were observed by light and immunofluorescence microscopy beforehand, and all of the patients’ diagnosis was confirmed based on current criteria. Later an experienced expert re-examined these biopsies, and all of the patients were eligible for consideration.

### Hyperspectral image collection

We performed hyperspectral imaging using a compound microscope system, where a pushbroom (line-scanning) imager SOC-710VP (Surface Optics Inc.) is employed combining with a microscope (Olympus CX31RTSF). The imaging system captures spatial size 696 × 520 with 128 spectral bands, covering the spectral range from 400 nm to 1000 nm with a spectral resolution of 4.69 nm. The heart of the HS imager consists of a spectral dispersion element and a 2-dimensional focal plane array (FPA) detector. In our system, the dispersive spectrometer is a diffraction grating where the incoming light produced from the microscope is separated into discrete wavelengths before being projected onto the detector. Next, the charge-coupled device (CCD) detector is activated to capture the intensity of the light at each pixel of the image using the HyperSpect™ operating software. For each patient, we randomly selected 2–3 glomeruli per slide, and then manually marked out every immune complex in the subepithelial area using the ENVI 14.0 software before exporting the data for further analysis.

### Image De-noising

**Fig. 2:**
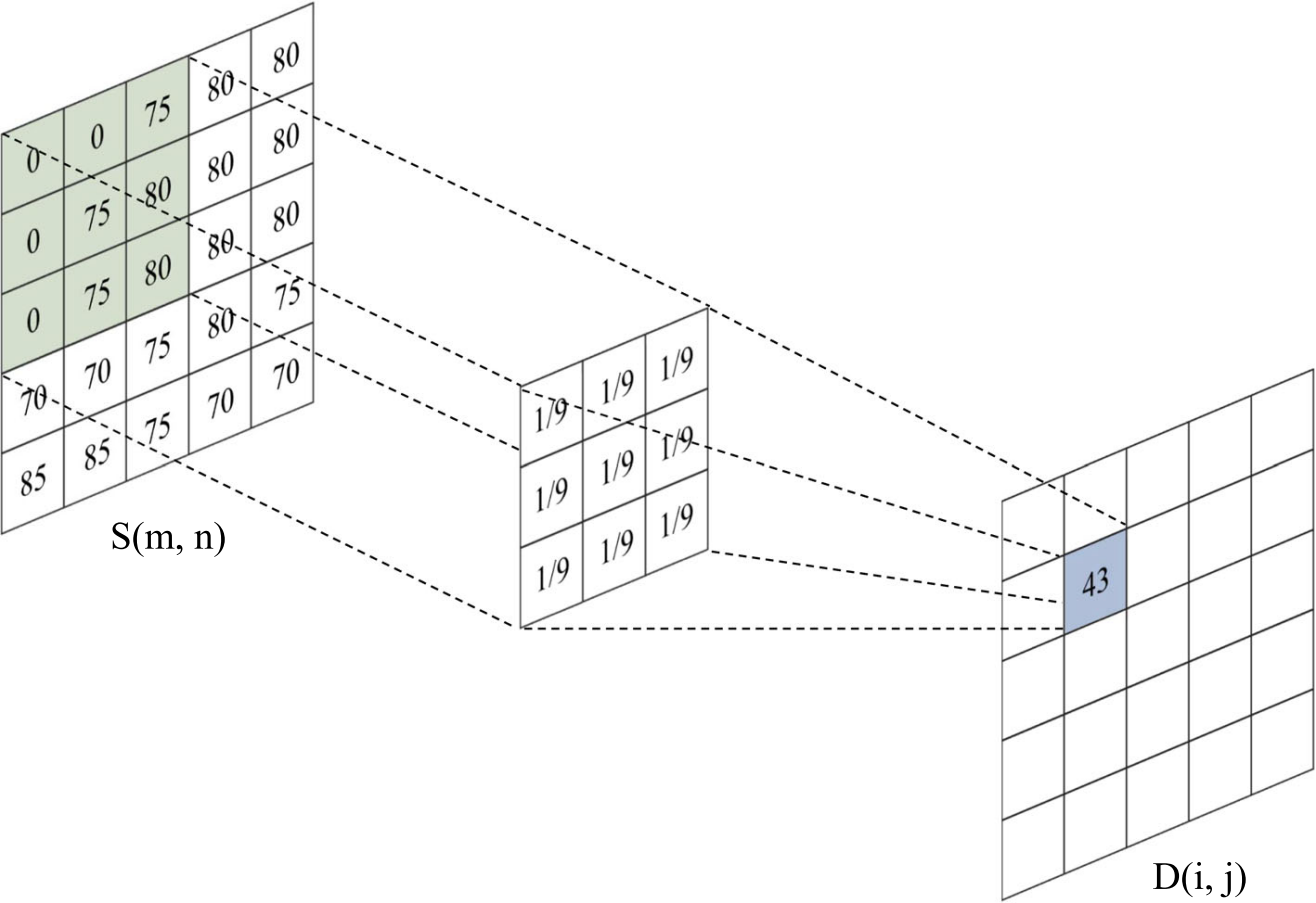
Concept of mean filtering. The value of each center pixel is replaced with the average value of all pixels inside the filtering window

Unprocessed HSI data usually contains high spectral noise generated by the imaging system. This noise can lead to undesirable effects. In order to remove the noise in the data, a mean filter was applied. Figure 2 shows the concept of mean filtering is to replace the center pixel with the average value of all of the pixels inside the local window, reducing the amount of intensity variation between one pixel and its neighbors. Here, the de-noising process is achieved via the following eq. (1). Where *D(i, j)* is the value of the center pixel in the filtering window, *S(m, n)* is the value of pixels in the window (i = 0,1 … H-1; j = 0,1 … W-1), and W and H represent the width and height of the filtering window, respectively.

1 $$ D\left(i,j\right)={\sum}_{\left(m,n\right)\in {R}_{i,j}}S\left(m,n\right)/ HW $$

**Fig. 3:**
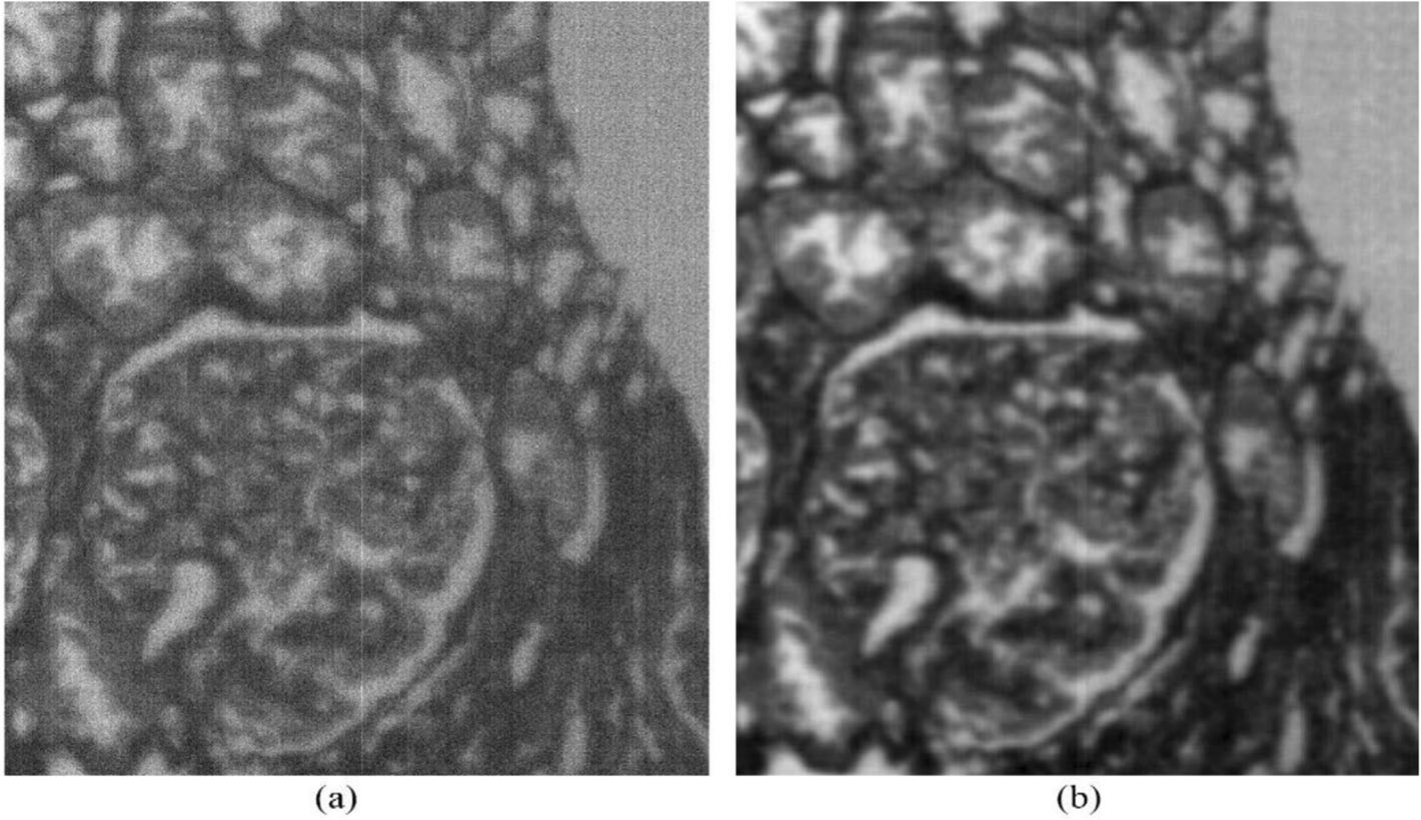
Panels (**a**) and (**b**) are before and after image de-noising of an HBV-MN patient’s glomeruli, the wavelength corresponds to the 10th channel is 442 nm

Figure 3 shows a glomerulus from the 10th channel of an HBV-MN patients’ HS image before and after de-noising, the wavelength corresponds to the 10th channel is 442 nm. The remarkable improvement confirms the effect of reducing the system noise of HSI data.

### Projection transformation

**Fig. 4:**
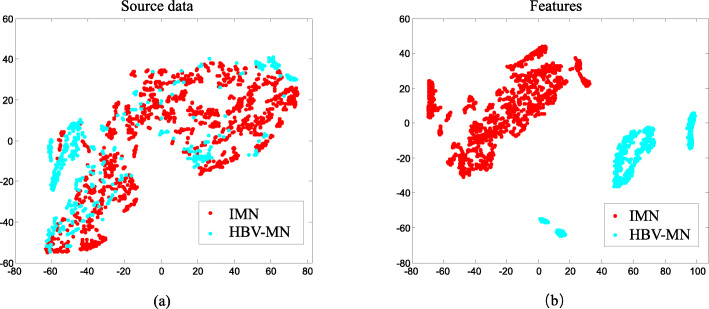
Panels (**a**) and (**b**) show the distribution of samples’ intrinsic features before and after the projection procedure

HSI data has hundreds of spectral information channels; hence, an essential step for utilizing HSI data is to reduce the redundant information in its spectral signature. Projection transformation is an advanced method for acquiring the maximum reduced subspace of a target without losing its essential information. Current projection transformation techniques include principal component analysis (PCA), independent component analysis (ICA), and Fisher’s linear discriminant analysis (LDA). However, a significant drawback of those techniques is that they all are only legitimate when the target data has a Gaussian distribution. In this study, we developed an alternative method named local Fisher’s discriminant analysis (LFDA). The typical LFDA projection is calculated by maximizing Fisher’s ratio and using these local scatter matrices. The biggest benefit of LFDA is that it can obtain good between-class separation in the feature subspace while preserving the within-class local structure [16]. It also integrates the advantages of both Fisher’s linear discriminant analysis (LDA) and locality-preserving projections (LPP) while bypassing the requirement for a Gaussian distribution [17–20]. Figure 4a and b are the before and after projection transformation feature distribution of one testing sample. The results exhibit the effectiveness of LFDA for seeking a subspace with maximum separability for features.

### Proposed deep learning framework

Following the image de-noising and projection transformation procedures mentioned above, we constructed a deep neural network (DNN) to extract and classify the intrinsic and high-level features of the different glomerular images [21]. More specifically, support vector machine (SVM), extreme learning machine (ELM) [22], Alexnet [23], Resnet20 [24], and VGG19 [25] were implied on the MN database with and without the pre-processing procedures to achieve the ultimate goal of formulating an MN identification architecture that can automatically distinguish HBV-MN from IMN. For the deep learning models: First, Leaky ReLU activation function is applied after each convolutional layer to solve the problem of gradient vanishing and accelerate the fitting speed; second, batch normalization (BN) strategy behind some convolutional layers is used as a regularizer to simplify the tuning process and lower initialization requirement; third, dropout technique is employed to avoid the over-fitting issue, and the dropout rate is set to 0.5. The proposed DL models were designed and developed using PyTorch.

The validation of the proposed DL framework was performed using leave-one-out cross-validation (LOOCV), where samples of the patients to be tested were extracted from the database before training the algorithm. This methodology guarantees the database for the training and testing process are strictly separated; it also proves the eligibility and consistency of comparing the performance between each method.

Here, we applied DNN to identify glomerular disease in microscopic hyperspectral images for the first time, and then verified and supplemented the outcome of immunofluorescence or light microscopy.

## Results

**Fig. 5:**
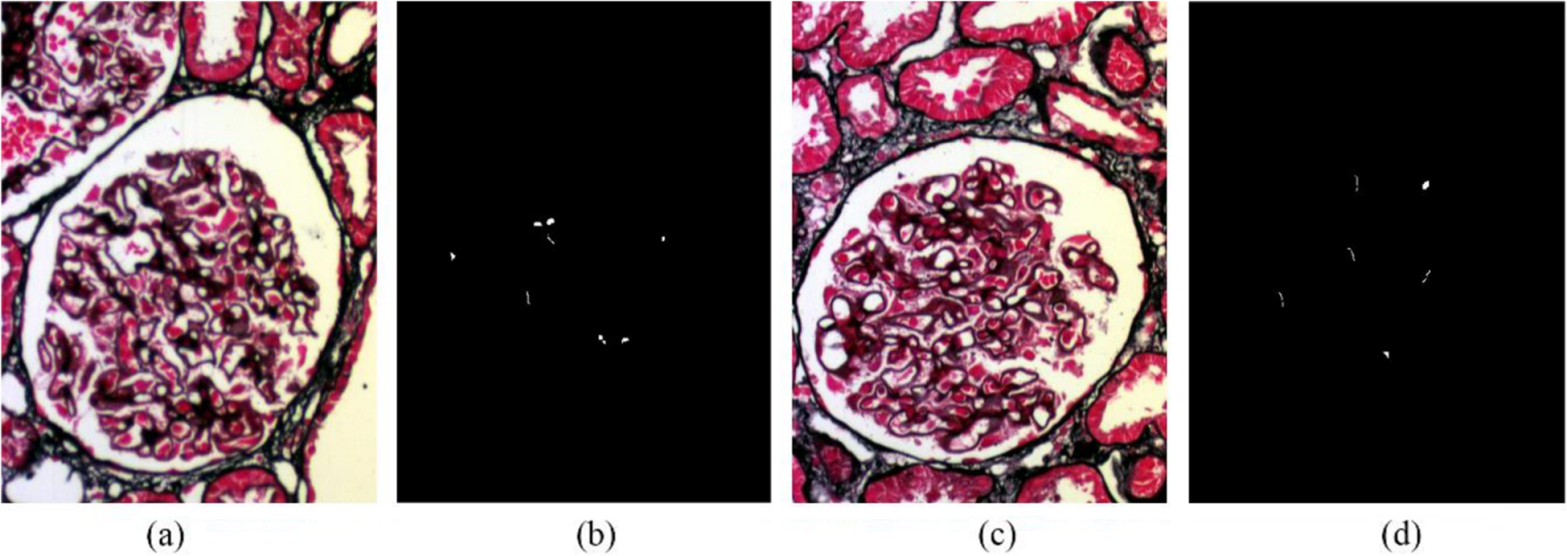
Panels (**a**) and (**c**) are images of an HBV-MN and IMN glomeruli; (**b**) and (**d**) are the corresponding ground truth maps with white pixels representing the marked out immune complexes

**Fig. 6:**
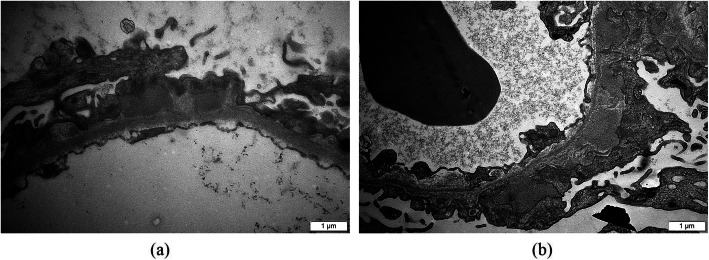
Panels (**a**) and (**b**) are pictures of an HBV-MN and an IMN immune complex deposition under 20,000× electron microscopy

We used the LDFA-DNN method mentioned above for a comparative evaluation of the hyperspectral image data of HBV-MN with IMN from renal biopsy tissue. We collected 30 HBV-MN images and 24 IMN images from 10 patients in each case of the MN database. Figure 5a and c shows an HBV-MN and an IMN sample under the light microscope; panels (b) and (d) highlight their immune complexes in the foreground, respectively. Figure 6 presents the corresponding immune complex deposition under an electron microscope. The side-by-side images of HBV-MN and IMN showed a high similarity of their biological features whether under light or electron microscope. Hence, we proceed with the pre-processing chain of de-noising and projection transformation to extract the intrinsic features for further classification.

After de-noising all of the original hyperspectral images, LFDA is applied to convert the massive HSI data into a reduced subspace (Fig. 4). Then, we conducted experiments to investigate the performance of VGG networks under different dimensions of the reduced subspace. Table 2 shows that LFDA obtains optimal classification performance for VGG networks when the dimension is 9.

**Table 2:** Comparison of overall accuracy (OA) and Kappa coefficients with different dimensions of reduced subspace for LFDA

Metrics	**5**	**9**	**13**	**17**	**21**
OA (%)	93.17	95.04	91.87	92.27	92.95
Kappa	0.8629	0.9006	0.8367	0.8458	0.8590

We also verified several patch sizes for the VGG network because of the significant impact it has on the DNNs performance. Table 3 suggests that 11 × 11 is the optimal size for the patches.

**Table 3:** The classification performance of various patch sizes

**Metrics**	**9 × 9**	**11 × 11**	**13 × 13**	**15 × 15**
OA (%)	93.13	95.04	92.43	94.36
Kappa	0.8629	0.9006	0.8485	0.8872

**Fig. 7:**
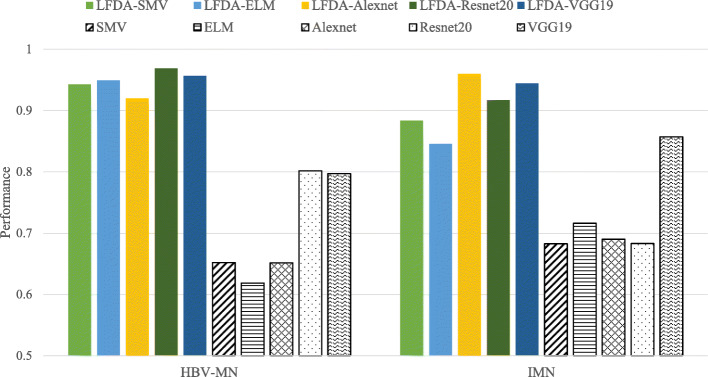
Comparison of the performance obtained from each approach

Table 4 shows the resulting accuracies of HBV-MN and IMN obtained by various approaches: SVM, ELM, Alexnet, Resnet20, VGG19 are implemented without preprocessing; LFDA-SVM, LFDA-ELM, LFDA-Alexnet, LFDA-Resnet20, and LFDA-VGG19 are implemented using features with filtering and reduced subspace. Accuracy is defined via the following eq. (2), where P is positive, N is negative, TP is true positive, and TN is true negative. The overall accuracy (OA), average accuracy (AA), and Kappa coefficient were computed to assess the result of different algorithms. The proposed framework LFDA-VGG19 achieved 94.45% accuracy for IMN and 95.67% accuracy for HBV-MN in binary classification, which improves the overall accuracy to 95.04% (12% more than the overall accuracy obtained by algorithms without pre-processing chain). Figure 7 shows the complete performance results obtained by each approach. Compared with conventional machine learning algorithms, deep learning models offer better performance in distinguishing HBV-MN from IMN. Furthermore, adding the de-noising and projection transformation significantly boosts LFDA-DNN’s performance; this validates the necessity of the pre-processing chain.

2 $$ Accuracy=\frac{TP+ TN}{P+N} $$

**Table 4:** The classification performance of the LFDA-DNN using the MN dataset

Comparisons	HBV-MN	IMN	OA (%)	AA (%)	Kappa
SVM	65.20	68.27	66.80	66.74	0.3347
ELM	61.85	71.62	66.94	66.74	0.3356
Alexnet	65.16	69.01	67.16	67.09	0.3418
Resnet20	80.17	68.31	73.99	74.24	0.4819
VGG19	79.70	85.72	82.84	82.71	0.6554
LFDA-SVM	94.28	88.35	91.19	91.31	0.8239
LFDA-ELM	94.92	84.58	89.54	89.75	0.7913
LFDA-Alexnet	92.00	96.00	94.09	94.00	0.8814
LFDA-Resnet20	96.88	91.72	94.19	94.30	0.8839
LFDA-VGG19	95.67	94.45	95.04	95.06	0.9006

**Fig. 8:**
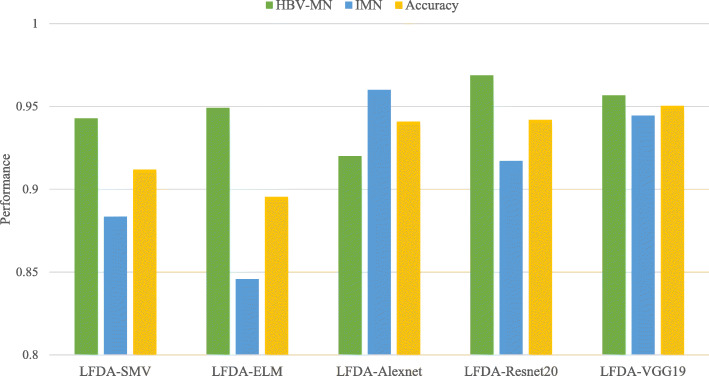
Overall accuracy of each LFDA-DNNS

Figure 8 shows that LFDA-VGG19 achieves outstanding performance for separate HBV-MN from IMN. Its overall accuracy improves by 3.85 and 5.50% higher than LFDA-SVM and LFDA-ELM, respectively. While LFDA-Resnet20 maintains the best one-on-one classification result for HBV-MN and LFDA-Alexnet for IMN, LFDA-VGG19 presents a more stable and balanced performance to identify and distinguish the two. Therefore LFDA-VGG19 is an ideal deep learning framework for this study.

For further research purposes, we also added 10 HBV-MN patients with negative serum HBV markers into the framework to compare with previous HBV-MN patients, and the sorting result came back as low separability between serum HBV markers positive HBV-MN patients and those without serum antigens/antibodies. This suggests that the hepatitis B virus could cause extrahepatic immune complex formation/deposition without serological evidence.

## Discussion

The WHO estimates that more than 257 million persons or 3.5% of the world population is living with chronic HBV infection [26]. Hepatitis B virus infection is a significant health problem in China. Previous case reports have associated chronic hepatitis B virus (HBV) infection with several types of glomerulonephritis (GN). Almost all of the morphological forms of renal disease, including membranoproliferative GN (MPGN) [27], mesangial proliferative GN, minimal change disease, focal glomerulosclerosis, and IgA nephropathy (IgAN) have been described [28]. The most common type of GN is membranous nephropathy, and the natural history of this condition is not clear [29]. By employing a hyperspectral imaging-based architecture consists of filtering, projection transformation, high-level feature extractor, and Softmax classification, we could identify HBV-MN and IMN cases using a novel and accurate technique.

Compared with conventional bidimensional images obtained by light microscopy, the hyperspectral imaging system provides superior resolution and sensitivity as well as the capability to simultaneously process multidimensional images [30, 31]. HSI was initially applied to fields such as remote sensing [32], archeology [33, 34], and drug identification [35, 36]. In recent years, the extension of HSI to the biomedical field has started to achieve promising results. In theory, compositions of different materials will present significant discrepancies in spectral curves [10]; therefore, curves of IMN and HBV-MN would be ready to distinguish due to their different immune complex components led by diverse pathogenesis. The hyperspectral analysis method we describe here demonstrates a wide spectral range and a high spectral resolution. The implement of LFDA for dimension reduction creates a subset of new features by seeking the best projection transformation using all the original spectral bands, this ensures the reduced feature space retains the useful information from the original data space without the useless information, such as spectral redundancy. These features, in combination with the de-noising procedures, ensure accuracy and reproducible imaging spectroscopy and spectrometry [9].

Medical hyperspectral imaging has been employed in several different areas such as blood vessel visualization enhancement [37], estimation of cholesterol levels [38], histopathological tissue analysis [11, 39], and identification of glioblastoma [40]. There are no studies regarding membranous nephropathy nor the distinction between IMN and HBV-MN. Potentially, the use of HSI in renal pathology field can improve the current situation in three ways: (1) HSI can extract the intrinsic features of target material in real-time, therefore rapid detection of HBV-MN and IMN on unstained slides could enhance the result of immunoelectron microscopy by binding the HBV antigen/antibody before fixation and embedding of the renal tissue; (2) With the refinement of current DL algorithms and the development of new frameworks, different glomerular diseases with immune complex formation such as IgA nephropathy (IgAN), membranoproliferative glomerulonephritis (MPGN), and dense deposit disease (DDD) can be diagnosed using HSI; (3) In traditional histological techniques, the degree of staining reaction between dye and tissue could be determined by several conditions such as the room temperature, pH of the solution, or the reaction time. Consequently, pathological images acquired from different facilities may vary depending on the circumstances. Meanwhile, visual assessment of these pathological images usually relies heavily on physicians’ experience level. HSI avoids the uncontrollable factors and the limitation of subjective judgment; it may also contribute to standardize the diagnosis criteria of certain diseases in the near future.

It is worth mentioning that our approach achieves over 95% accuracy in classification. The result demonstrates the ability of the proposed framework to achieve high performance in the correct detection of renal diseases with immune complex formation, which is ideal in the design of a decision support system for pathological diagnosis.

We recognize several limitations to this study. First, given the relatively small number of patients enrolled in the MN dataset, larger scale studies are warranted in the future to prospectively validate the effectiveness of the deep learning framework. Also, multi-class classification should be performed on different pathological types of glomerular disease to fully develop the computer-aided diagnosis system. Second, there are no standard protocols for image calibration, data formation/validation, etc., to make the proposed framework compatible. Finally, the analysis mechanism identifies what property of immune complex deposition related to the spectral signatures is not clear [9]. Further experiments should be performed to interpret the implications of these spectral features and their association with the pathogenesis of the disease.

## Conclusion

In this study, we propose hyperspectral analysis as a new method for the characterization and distinction of HBV-MN from IMN, especially for cases where the discrimination is not ideal with light microscopy. The outcomes in this preliminary work demonstrate the feasibility of using hyperspectral imaging-based LFDA-DNN architecture as an alternative method for the identification of HBV-MN and its potential use as a supplementary tool for renal pathology.

## Data Availability

The dataset used and/or analyzed during the current study are available from the corresponding author on reasonable request.

## Abbreviations

MN: Membranous nephropathy
IMN: Idiopathic membranous nephropathy
HBV: Hepatitis B virus
DL: Deep learning
HSI: Hyperspectral imaging
DNN: Deep neural network
LFDA: Local Fisher’s discriminant analysis
SMV: Support vector machine
ELM: Extreme learning machine
